# Quantitative angiographic markers associated with symptomatic radiation-induced changes in supratentorial brain arteriovenous malformations after radiosurgery

**DOI:** 10.1007/s00330-025-11884-8

**Published:** 2025-07-31

**Authors:** Wei-Lun Kao, Chung-Jung Lin, Jing Kai Loo, Huai-Che Yang, Cheng-Chia Lee, Sanford PC. Hsu, Hong-Jen Chiou, Feng-Chi Chang, Kang-Du Liu, Yong-Sin Hu

**Affiliations:** 1 Department of Radiology, Taipei Veterans General Hospital, Taipei City, Taiwan; 2 School of Medicine, National Yang Ming Chiao Tung University, Taipei, Taiwan; 3 Department of Radiology, Shuang Ho Hospital, Taipei Medical University, Taipei, Taiwan; 4 Department of Neurosurgery, Neurological Institute, Taipei Veterans General Hospital, Taipei, Taiwan; 5 Department of Radiology, Taipei Hospital, Ministry of Health and Welfare, New Taipei, Taiwan

**Keywords:** Angiography, Brain arteriovenous malformations, Hemodynamics, Radiosurgery, Radiation-induced changes

## Abstract

**Objectives:**

Radiation-induced changes (RICs) may cause neurological deficits in patients with brain arteriovenous malformations (BAVMs) after radiosurgery. The present study investigated quantitative angiographic markers contributing to symptomatic RICs.

**Materials and methods:**

A total of 131 patients with supratentorial BAVMs who had not received prior treatment and underwent radiosurgery between 2011 and 2020 were included. Patients completed ≥ 24 months of MRI and clinical follow-up. MRIs and angiograms taken before radiosurgery were analyzed for morphological characteristics and quantitative angiographic parameters. Symptomatic RICs were defined as neurological symptoms attributed to RICs. The vein-artery (VA) ratio was defined as the sum of all draining vein diameters divided by the sum of all supplying artery diameters. The modified cerebral circulation time (mCCT) was defined as the interval between the bolus arrival time of the ipsilateral cavernous internal carotid artery and the parietal vein. Logistic regression models were used to evaluate associations between these markers and symptomatic RICs.

**Results:**

Symptomatic RICs developed in 27 (20.6%) of 131 patients. Nine patients with symptomatic RICs were hospitalized. Multivariable analysis revealed that a lower VA ratio and shorter mCCT were independently associated with symptomatic RICs. Furthermore, the quantitative angiographic model exhibited a higher performance in association with symptomatic RICs than the angioarchitectural model did.

**Conclusion:**

A lower VA ratio and shorter mCCT were quantitative angiographic markers of venous outflow impairment and high blood flow of BAVMs, respectively. These markers may quantify the hemodynamic effect that contributes to symptomatic RICs development in patients with BAVMs after radiosurgery.

**Key Points:**

***Question*** *Can quantitative angiographic markers be used to evaluate the risks of symptomatic radiation-induced changes (RICs) in patients with brain arteriovenous malformations (BAVMs) after radiosurgery?*

***Findings*** *BAVMs with a lower vein-artery ratio or shorter modified cerebral circulation time were associated with higher risks of symptomatic RICs development.*

***Clinical relevance*** *Quantification of the BAVM hemodynamic effect that contributes to symptomatic RICs development may help therapeutic decision-making for patients with BAVMs after radiosurgery.*

**Graphical Abstract:**

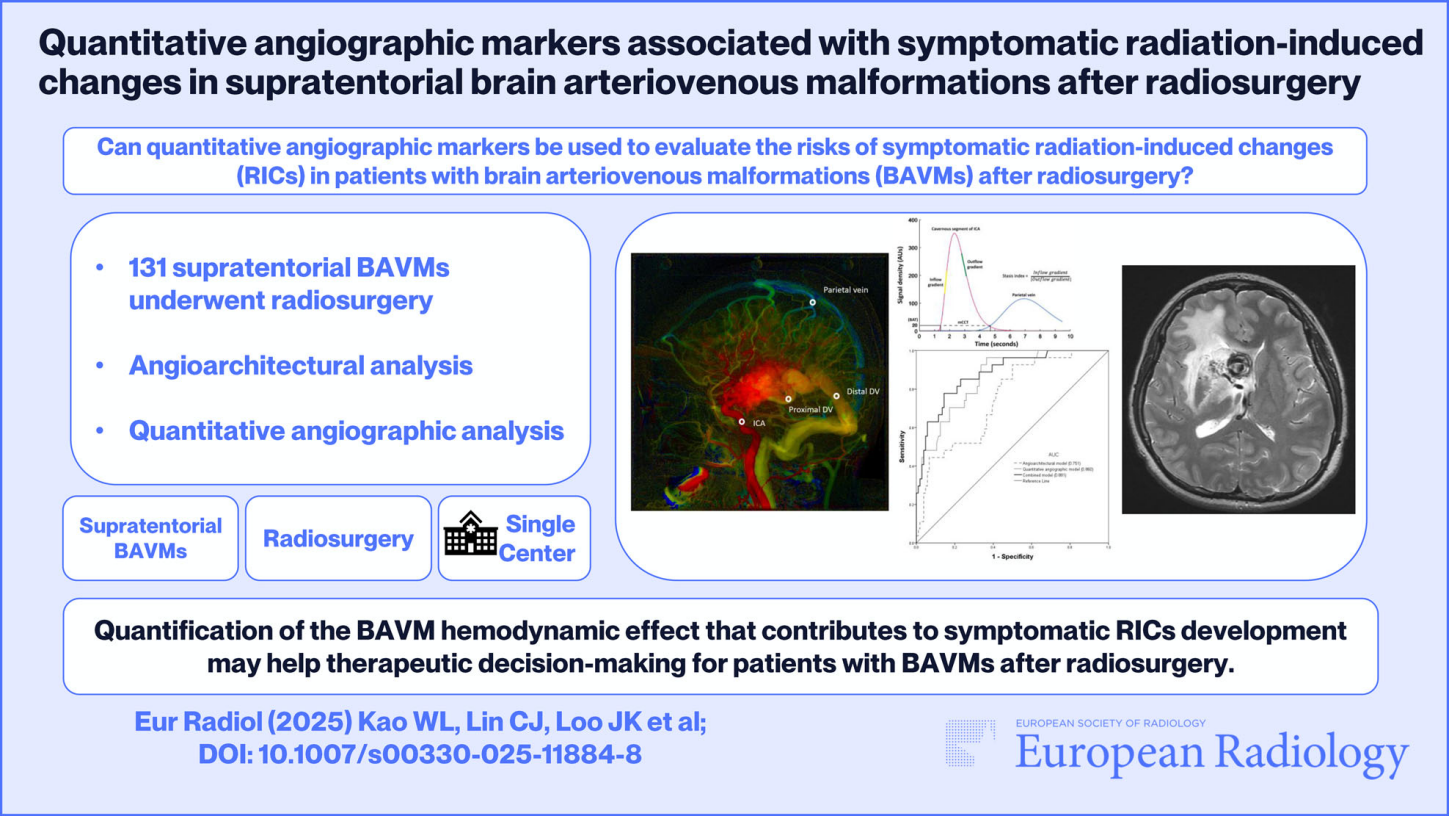

## Introduction

Brain arteriovenous malformations (BAVMs) are rare vascular lesions found in approximately 18 of every 100,000 individuals [1]. BAVMs consist of abnormal connections between cerebral arteries and veins, typically involving an intervening nidus, and may cause intracranial hemorrhage, headaches, seizures, and neurological deficits [1]. Gamma Knife radiosurgery (GKRS) has been proven effective as treatment for BAVMs [2, 3]. In a meta-analysis by Ilyas et al, GKRS achieved complete obliteration (CO) in 68% of unruptured BAVMs at last follow-up [4].

Notably, post-treatment complications may result from radiation-induced changes (RICs) after GKRS, potentially causing catastrophic neurological deficits [5]. Another meta-analysis by Ilyas et al revealed that GKRS caused radiologic and symptomatic RICs in 35.5% and 9.2% of patients with BAVMs, respectively [6]. RICs can be identified on the basis of new or increased perinidal high T2 signal intensity on MRI images, with such intensity peaking approximately 16 months after GKRS [7, 8]. The mechanisms leading to RICs include brain tissue damage caused by direct radiation effects, parenchymal edema caused by occlusive hyperemia, and neoangiogenesis secondary to perinidal hypoxemia [8–10]. The risk factors of symptomatic RICs include larger nidal volume, lesions at eloquent and deep locations, higher Spetzler–Martin grade, unruptured BAVMs, higher RICs grade, and higher margin dose [7, 11–14]. Prior BAVM hemorrhage causes perinidal changes, including gliosis and hemosiderin deposition, and may reduce radiation effects on the surrounding parenchyma against neurological injury [7, 13, 14].

Quantitative methods have been used to predict the outcomes of patients with BAVMs after GKRS. In one study, compact unruptured BAVMs, determined according to the compactness index, exhibited a higher CO rate and a lower risk of RICs than diffuse BAVMs did [15]. In addition, Takeda et al measured the blood flow of the largest feeding artery by using time-averaged 3-dimensional flow MRI, discovering a significantly higher degree of flow reduction in BAVMs that achieved CO within 3 years after GKRS [16]. Furthermore, a lower vein-artery (VA) ratio, as measured using digital subtraction angiography (DSA), was suggested to be an independent predictor of RICs following GKRS [9]. However, given that RICs are multifactorial, these findings require validation in a larger sample of patients with BAVMs. Quantitative DSA (QDSA) has been used to identify BAVMs with stagnant venous flow, which can contribute to CO after GKRS, whereas high flow has been associated with resistance to CO [17, 18]. Therefore, the present study explored quantitative angiographic markers associated with symptomatic RICs in patients with BAVMs after undergoing GKRS.

## Materials and methods

### Patient selection and clinical characteristics

**Fig. 1:**
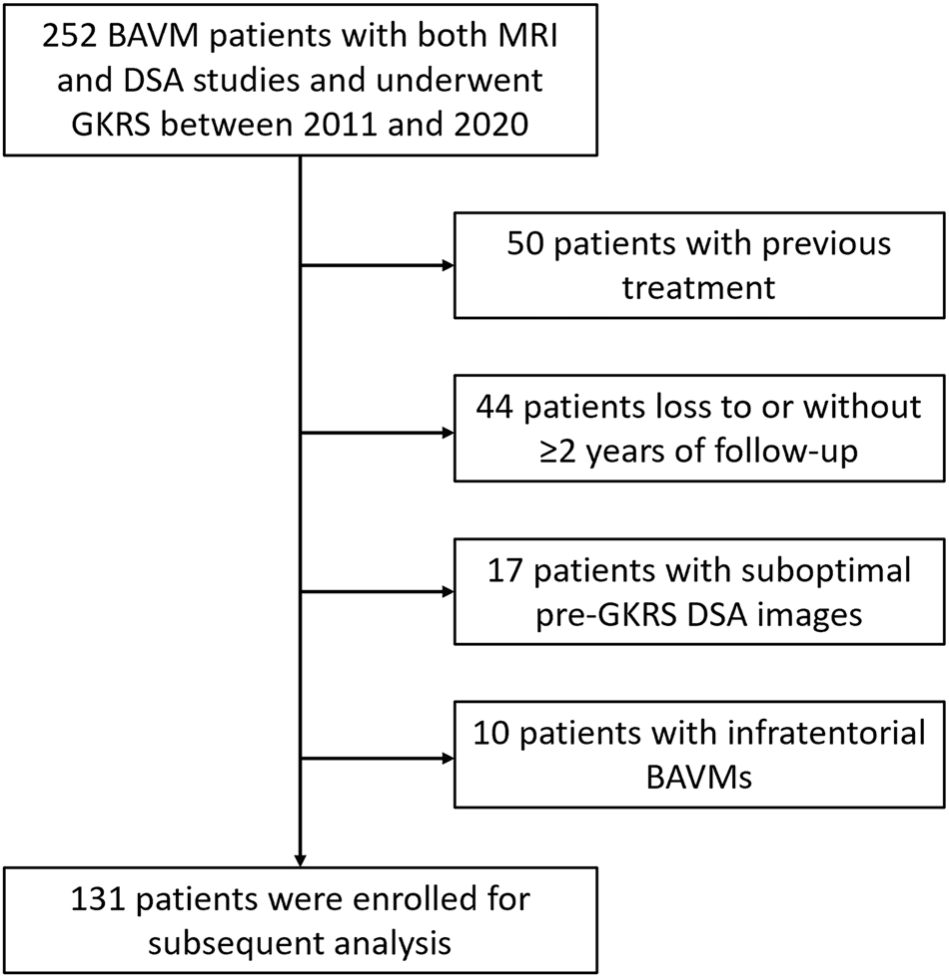
Patient selection flowchart. BAVM, brain arteriovenous malformation; DSA, digital subtraction angiography; GKRS, Gamma Knife radiosurgery

This manuscript follows TRIPOD reporting guidelines [19]. We conducted a retrospective study of 252 patients with BAVMs who underwent GKRS at our institution between 2011 and 2020. All patients had undergone both MRI and DSA diagnostic procedures before treatment. The exclusion criteria included a history of treatment for BAVMs (*n* = 50), such as embolization, radiosurgery, or microsurgery; MRI and clinical follow-up less than 2 years after GKRS (*n* = 44); inadequate quality of pre-GKRS DSA images for subsequent QDSA analysis (*n* = 17); and presence of infratentorial BAVMs (*n* = 10). Patients previously treated for BAVMs were excluded because these treatments cause permanent changes to the surrounding brain parenchyma. A total of 131 patients were included in subsequent analyses (Fig. 1). Demographic information (age and sex) and clinical presentations (such as headache, seizure, neurologic deficits, and hemorrhage) were collected through chart reviews. The local institutional review board waived the need for consent in consideration of the retrospective nature of the study.

### MRI and DSA acquisition

MRI was performed using 1.5-T scanners following a protocol that included axial fast spin-echo T2-weighted imaging with 3-mm-thick slices and axial unenhanced 3-dimensional time-of-flight MRA with 1.5-mm-thick slices. DSA examination was conducted for all patients by using a standardized protocol in the angiosuites, Artis zee (2011–2019) and Artis Q (2019–2020) (Siemens Healthcare). After a 4-Fr angiocatheter was placed in the ipsilateral common carotid artery at the C4 vertebral level, a bolus of 12 to 14 mL iodine contrast medium was administered using a power injector within 1.5 s (Liebel-Flarsheim Angiomat, Illumena). The image acquisition protocol involved obtaining 7.5 frames/s for the first 5 s, followed by 4 frames/s for 3 s, 3 frames/s for 2 s, and 2 frames/s for the final 2 s [18, 20].

### Stereotactic radiosurgery and clinical follow-up protocol

Stereotactic radiosurgery was performed using Gamma Knife (Elekta AB) models 4C (2011 to 2012) and Perfexion (2012 to 2020). Stereotactic MRI and DSA were conducted after a Leksell model G (Elekta AB) stereotactic frame was fixed to the patient’s head. MRI and DSA images were integrated for BAVM nidus delineation to determine GKRS target and dose [8, 17]. After GKRS, patients completed outpatient follow-up for neurological assessments at least every 3 months and underwent MRI every 6 months.

### Follow-up MRI findings and outcomes

**Fig. 2:**
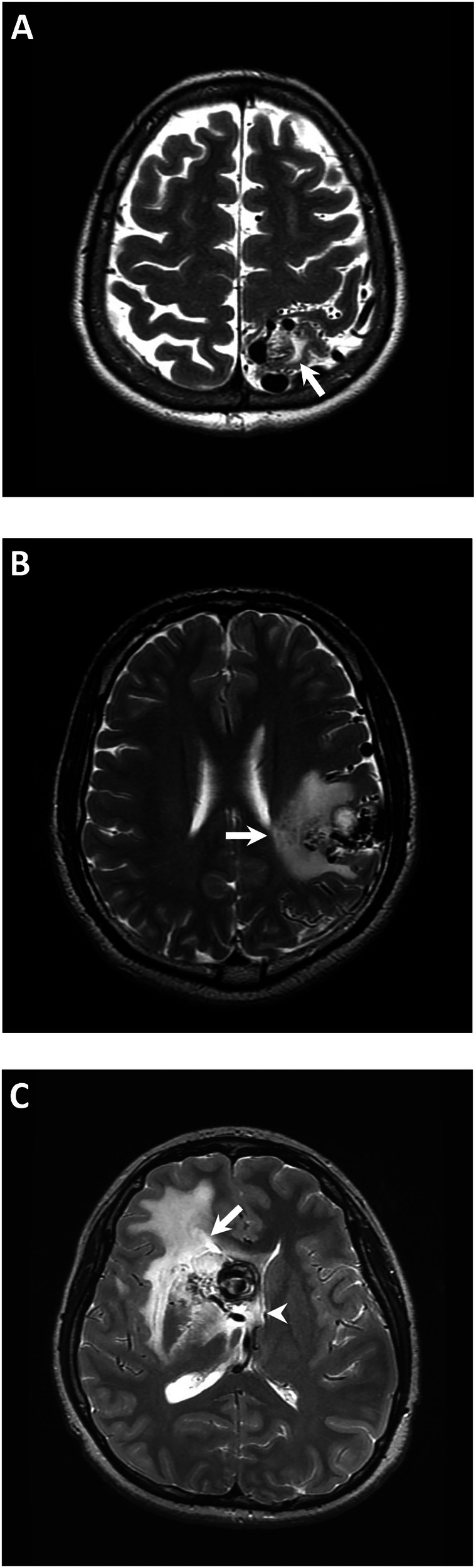
Examples of the three grades of radiation-induced changes (RICs) severity on axial T2-weighted images. **A** Grade I (mild) RICs at left parietal lobe with increased T2 hyperintensity < 1 cm (arrow) and no mass effect. **B** Grade II (moderate) RICs at left posterior frontal lobe with increased T2 hyperintensity ≥ 1 cm and mild ventricular compression (arrow). **C** Grade III (severe) RICs at right frontal lobe with severe mass effect, marked ventricular compression (arrow), and cerebral midline shift (arrowhead)

Follow-up MRI was conducted to determine whether RICs were present, as indicated by increased or newly developed perinidal T2 high signal intensity after GKRS [5, 8, 9]. RICs severity was evaluated according to the grading system proposed by Yen et al, with grade I (mild) constituting increased T2 hyperintensity < 1 cm, grade II (moderate) constituting increased T2 hyperintensity ≥ 1 cm and ventricular compression or sulcus effacement, and grade III (severe) constituting cerebral midline shift (Fig. 2) [7]. Symptomatic RICs were defined as the development of neurological symptoms, such as headache, seizure, or neurological deficits, attributable to RICs [8, 9]. The timing of symptomatic RICs development following GKRS and subsequent management strategies were documented. CO was confirmed through MRI based on an absence of flow-related enhancement on MRA and flow voids on T2-weighted images or through DSA based on the disappearance of abnormal arteriovenous shunting [8, 17, 18].

### BAVM and angioarchitectural features

In addition to the location, eloquence, nidal volume, and Spetzler–Martin grade of BAVMs, we investigated angioarchitectural features with reference to previous studies. These features were defined in accordance with the reporting terminology provided by the American Society of Interventional and Therapeutic Neuroradiology [8, 17, 18, 20–22]. Flow-related aneurysm was defined as an aneurysm arising from an artery supplying the nidus [8, 17, 18, 20, 22]. Intranidal aneurysm was defined as an aneurysm located within the boundaries of or contiguously with the nidus [8, 17, 18, 22]. Neoangiogenesis was defined as the presence of dilated perinidal arteries or the recruitment of leptomeningeal collateral arteries due to arterial steal caused by a high-flow nidus [8, 17, 18, 20]. Deep venous drainage was defined as drainage through internal cerebral or precentral cerebral veins [8, 17, 18, 21, 22]. Venous stenosis was defined as the presence of a main drainer with a focal diameter 50% smaller than the adjacent venous diameter [8, 17, 18, 20, 22]. Venous ectasia was defined as a diameter change of more than 200% in the draining veins [8, 20]. Pseudophlebitic pattern was defined as tortuous and dilated collateral veins that drained brain parenchyma in the venous phase [8, 17, 18, 20]. BAVMs with superior sagittal sinus (SSS) drainage or parietal cortical drainage were recorded.

### Quantitative angiographic measurements

**Fig. 3:**
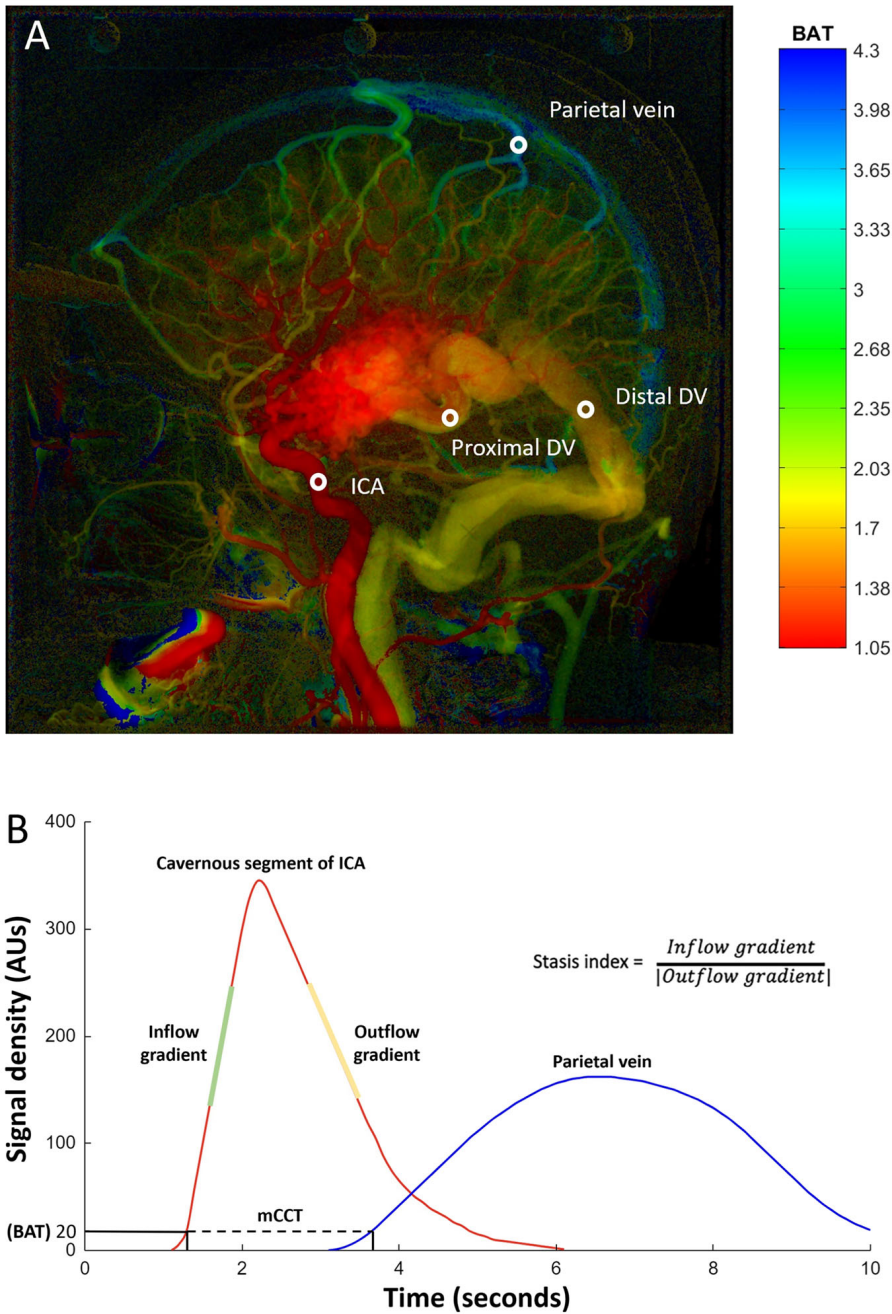
Illustration of quantitative DSA (QDSA) analysis for BAVMs. **A** Lateral view of QDSA images color-coded on the basis of bolus arrival time (BAT, seconds). Regions of interest (ROIs) included the cavernous segment of the internal carotid artery (ICA), proximal and distal portions of draining veins (DVs), and parietal vein outlet. **B** QDSA parameters and time-density curve. BAT was defined as the first time point at which the density exceeded 20 arbitrary units (AUs). Modified cerebral circulation time (mCCT) was defined as the BAT difference between ROIs of the cavernous ICA and parietal vein. Inflow and outflow gradients were defined as the largest and smallest slopes, respectively, of all fitted linear functions. The stasis index was defined as the inflow gradient divided by the absolute value of the outflow gradient

The VA ratio, defined as the sum of all draining vein diameters divided by the sum of all supplying artery diameters (see Electronic Supplementary Material) [18]. Anteroposterior and lateral views of DSA images were both used to determine the diameters of individual draining veins and supplying arteries. The narrowest portion of each draining vein was measured if caliber change was observed between the proximal and distal segments. The supplying arteries were defined as the trunk arteries giving rise to the arterial pedicles feeding the BAVM nidus. Figure 3 illustrates the QDSA analysis, which involved postprocessing analysis of the lateral view of the ipsilateral common carotid angiography [8, 17, 18, 20, 22]. The regions of interest (ROIs) included the cavernous segment of the internal carotid artery (ICA) supplying the BAVM, proximal and distal portions of the draining veins, and parietal vein outlet. The time-density curves of the selected ROIs were fitted with a gamma variate function using a customized program (MATLAB, MathWorks). Bolus arrival time (BAT) was defined as the first time point at which density exceeded 20 arbitrary units [18, 20, 22]. Transnidal time was defined as the BAT difference between the cavernous ICA and the proximal portion of the main draining vein [20]. Transvenous time was defined as the BAT difference between the proximal and distal portions of the main draining vein [20]. Modified cerebral circulation time (mCCT) was defined as the BAT difference between the cavernous ICA and the parietal vein outlet [18, 20]. Flow gradients were derived using the linear least-squares method, which involved sequentially fitting four consecutive temporal data points in the gamma variate function. The inflow and outflow gradients were defined as the largest and smallest slopes, respectively, among all fitted linear functions. The stasis index was defined as the inflow gradient divided by the absolute value of the outflow gradient, indicating the degree of venous outflow stagnation [17, 22]. The average stasis index of all distal draining veins was used for subsequent calculations. All angioarchitectural features and quantitative angiographic measurements were analyzed by two neuroradiologists blinded to the clinical data, who respectively had 18 and 9 years of neuroimaging experience. Consensus interpretations and average measurements were used for the subsequent analysis.

### Statistical analysis

Statistical analysis was performed using SPSS version 24.0 (SPSS, IBM). For categorical and continuous variables, results were presented as numbers (percentages) and median values (interquartile ranges), respectively. Fisher’s exact test was used to compare differences for categorical variables, and the Mann–Whitney *U* test was used for continuous variables. Spearman correlation was used to determine correlations among continuous variables. Following univariable Firth logistic regression analysis, variables with *p* < 0.10 were included in subsequent multivariable analysis. Receiver operating characteristic (ROC) analysis was used to determine VA ratio and mCCT cutoff values on the basis of the Youden index. Angioarchitectural and quantitative angiographic models were built to compare their performance in association with symptomatic RICs. The DeLong test was used to compare the area under the ROC curve between models. The Kaplan–Meier method and log-rank tests were used to determine differences in the time to initial development of symptomatic RICs. The statistical significance level was set at *p* < 0.05.

## Results

### Patient characteristics and outcomes

The demographic data, clinical presentations, outcomes, BAVM characteristics, GKRS parameters, angioarchitectural features, and quantitative angiographic parameters for all included patients are summarized in Table 1. Of the 131 included patients, 58 (44.3%) were female, and the median age at the time of GKRS was 32 years (interquartile range, 23 to 45 years). Clinical presentations included headache in 63 patients (48.1%), seizure in 45 (34.4%), neurological deficits in 62 (47.3%), and hemorrhage in 59 (45%). No sex or age differences were observed between the groups with symptomatic RICs, asymptomatic RICs, and no RICs.

**Table 1:** Demographics, clinical presentations, outcomes, BAVM characteristics, GKRS parameters, angioarchitectural features, and quantitative angiographic parameters

Variable	Value (interquartile range)				*p*-value		
	Overall (*n* = 131)	With symptomatic RICs (*n* = 27)	With asymptomatic RICs (*n* = 79)	Without RICs (*n* = 25)	Symptomatic RICs vs asymptomatic RICs	Symptomatic RICs vs without RICs	Asymptomatic RICs vs without RICs
Median age at GKRS (years)	32 (23–45)	32 (19–47)	32 (23–44)	30 (24–48)	0.99	0.96	0.95
Female (%)	58 (44.3)	14 (51.9)	31 (39.2)	13 (52)	0.27	1.00	0.35
Clinical presentations (%)							
Headache	63 (48.1)	15 (55.6)	37 (46.8)	11 (44)	0.51	0.58	0.82
Seizure	45 (34.4)	11 (40.7)	29 (36.7)	5 (20)	0.82	0.14	0.15
Neurological deficit	62 (47.3)	17 (63)	32 (40.5)	13 (52)	0.048*	0.58	0.36
Hemorrhage	59 (45)	8 (29.6)	32 (40.5)	19 (76)	0.36	0.001*	0.003*
Follow-up							
Median clinical follow-up (months)	58 (34–83)	56 (36–75)	59 (36–81)	57 (31–99)	0.60	0.76	0.95
Median neuroimaging follow-up (months)	55 (34–80)	51 (34–69)	58 (35–80)	44 (29–88)	0.43	0.99	0.56
Outcomes							
Peak RICs grade (%)					0.003*	…	…
No	25 (19.1)	…	…	25 (100)	…	…	…
Grade I	22 (16.8)	4 (14.8)	18 (22.8)	…	…	…	…
Grade II	78 (59.5)	18 (66.7)	60 (75.9)	…	…	…	…
Grade III	6 (4.6)	5 (18.5)	1 (1.3)	…	…	…	…
Symptomatic RICs (%)	27 (20.6)	27 (100)	…	…	…	…	…
Headache	13 (9.9)	13 (48.1)	…	…	…	…	…
Seizure	8 (6.1)	8 (29.6)	…	…	…	…	…
Neurological deficits	10 (7.6)	10 (37)	…	…	…	…	…
Hospitalization required	9 (6.9)	9 (33.3)	…	…	…	…	…
Increased perinidal T2 hyperintensity at last follow-up (%)	69 (52.7)	17 (63)	52 (65.8)	…	0.82	…	…
Complete obliteration (%)	99 (75.6)	18 (66.7)	59 (74.7)	22 (88)	0.46	0.10	0.27
Development of hemorrhage after GKRS (%)	3 (2.3)	0 (0)	2 (2.5)	1 (4)	1.00	0.48	0.57
BAVM Locations (%)					0.10	0.06	0.17
Lobar (frontal, temporal, parietal, occipital)	107 (81.7)	21 (77.8)	66 (83.5)	20 (80)	…	…	…
Thalamus, basal ganglia	14 (10.7)	5 (18.5)	5 (6.3)	4 (16)	…	…	…
Corpus callosum	7 (5.3)	0 (0)	7 (8.9)	0 (0)	…	…	…
More than 2 locations	3 (2.3)	1 (3.7)	1 (1.3)	1 (4)	…	…	…
Deep location (%)	32 (24.4)	7 (25.9)	18 (22.8)	7 (28)	0.80	1.00	0.60
Eloquence (%)	88 (67.2)	18 (66.7)	53 (67.1)	17 (68)	1.00	1.00	1.00
Median BAVM volume (cm^3^)	7.52 (3.42–13.92)	12.73 (7.1–18.7)	8.8 (4.16–13.81)	1.21 (0.41–3.92)	0.03*	< 0.001*	< 0.001*
Volume > 5 cm^3^	82 (62.6)	22 (81.5)	56 (70.9)	4 (16)	0.32	< 0.001*	< 0.001*
Spetzler–Martin grade (%)					0.23	0.53	0.07
I	14 (10.7)	3 (11.1)	5 (6.3)	6 (24)	…	…	…
II	59 (45)	9 (33.3)	40 (50.6)	10 (40)	…	…	…
III	38 (29)	10 (37)	21 (26.6)	7 (28)	…	…	…
IV	19 (14.5)	4 (14.8)	13 (16.5)	2 (8)	…	…	…
V	1 (0.8)	1 (3.7)	0 (0)	0 (0)	…	…	…
GKRS treatment parameters					…	…	…
Median radiation volume (cm3)	9.7 (4.44–18.05)	15.42 (8.8–24.4)	11.8 (6–18.7)	2.2 (0.6–5.13)	0.06	< 0.001*	< 0.001*
Median margin dose (Gy)	18 (17.5–18)	18 (17.5–18)	18 (17.5–18)	18.5 (18–20)	0.25	0.002*	0.006*
Median max dose (Gy)	31.58 (30–33.04)	30.91 (29.46–32.7)	31.58 (30–33.3)	32.3 (30.9–33.3)	0.23	0.07	0.37
Median isodose line (%)	57 (54–60)	58 (55–60)	57 (54–59)	56 (54–60)	0.38	0.89	0.53
Angioarchitectural features (%)							
Flow-related aneurysm	6 (4.6)	2 (7.4)	3 (3.8)	1 (4)	0.60	1.00	1.00
Intranidal aneurysm	17 (13)	6 (22.2)	11 (13.9)	0 (0)	0.37	0.02*	0.06
Neoangiogenesis	58 (44.3)	17 (63)	38 (48.1)	3 (12)	0.27	< 0.001*	0.001*
Deep venous drainage	50 (38.2)	8 (29.6)	31 (39.2)	11 (44)	0.49	0.39	0.82
Venous stenosis	25 (19.1)	4 (14.8)	14 (17.7)	7 (28)	1.00	0.32	0.27
Venous ectasia	27 (20.6)	7 (25.9)	17 (21.5)	3 (12)	0.61	0.30	0.39
Pseudophlebitic pattern	22 (16.8)	9 (33.3)	11 (13.9)	2 (8)	0.04*	0.04*	0.73
Median number of draining vein(s)	2 (1–3)	2 (1–3)	2 (1–3)	1 (1–2)	0.53	0.01*	0.007*
SSS drainage	83 (63.4)	22 (81.5)	53 (67.1)	8 (32)	0.22	< 0.001*	0.003*
Parietal cortical drainage	50 (38.2)	12 (44.4)	33 (41.8)	5 (20)	0.83	0.08	0.06
Quantitative angiographic parameters							
Median VA ratio	1.96 (1.33–3.18)	1.37 (1.07–1.82)	2.19 (1.54–3.46)	1.88 (1.24–3.44)	< 0.001*	0.03*	0.23
Median transnidal time (s)	0.65 (0.5–0.8)	0.72 (0.54–0.87)	0.65 (0.53–0.75)	0.64 (0.39–0.93)	0.37	0.44	0.50
Median transvenous time (s)	0.37 (0.2–0.67)	0.44 (0.28–0.67)	0.33 (0.17–0.7)	0.38 (0.23–0.71)	0.37	0.85	0.63
Median mCCT (s)	2.39 (1.8–2.9)	1.95 (1.23–2.73)	2.39 (1.88–2.91)	2.65 (2.17–3.06)	0.02*	0.006*	0.28
Median average stasis index of distal draining veins	1.8 (1.6–2.24)	1.69 (1.56–1.97)	1.83 (1.61–2.35)	1.81 (1.68–2.54)	0.11	0.06	0.40

*BAVM* brain arteriovenous malformation, *GKRS* Gamma Knife radiosurgery, *RICs* radiation-induced changes, *SSS* superior sagittal sinus, *VA ratio* vein-artery ratio, *mCCT* modified cerebral circulation time

* Statistically significant

The median clinical and neuroimaging follow-up durations after GKRS were 58 months (34 to 83 months) and 55 months (34 to 80 months), respectively. RICs were detected in 106 patients (80.9%), among whom 27 (20.6%) developed neurological symptoms attributable to RICs. The frequency of grade II and III RICs did not differ significantly between patients with symptomatic and asymptomatic RICs (85.2% vs. 77.2%, *p* = 0.38). Among the 27 patients with symptomatic RICs, 13 (48.1%) presented with headache, 8 (29.6%) with seizure, and 10 (37%) with neurological deficits. Nine of these patients (33.3%) required hospitalization. The median time at which symptomatic RICs appeared after GKRS was 6 months (1 to 12 months). Twenty-one patients (77.8%) received steroids, and three (11.1%) received bevacizumab. Of the 106 patients with RICs, these changes persisted at the last follow-up MRI for 69 (65.1%). CO was achieved through GKRS in 99 patients (75.6%) following a median latency period of 32 months (28 to 40 months). The CO rates did not differ significantly between the three patient groups. Only three patients (2.3%) developed a hemorrhage after GKRS, but none of these patients exhibited symptomatic RICs.

### BAVM characteristics, GKRS parameters, angioarchitecture, and quantitative angiographic parameters

The median BAVM volume was significantly larger in patients with symptomatic RICs than in those with asymptomatic RICs (12.73 cm^3^ vs. 8.8 cm^3^, *p* = 0.03) or no RICs (12.73 cm^3^ vs. 1.21 cm^3^, *p* < 0.001). A total of 107 patients (81.7%) had lobar BAVMs, 32 (24.4%) had deep-seated BAVMs, and 88 (67.2%) had eloquent BAVMs. The median margin dose of GKRS was 18 Gy (17.5 to 18 Gy). Intranidal aneurysm (22.2% vs. 0%, *p* = 0.02), neoangiogenesis (63% vs. 12%, *p* < 0.001), pseudophlebitic pattern (33.3% vs. 8%, *p* = 0.04), and SSS drainage (81.5% vs. 32%, *p* < 0.001) were more frequently observed in patients with symptomatic RICs than in those without RICs. Median VA ratio and mCCT values were significantly smaller for patients with symptomatic RICs compared to those who had asymptomatic or no RICs. The VA ratio exhibited very weak correlations with BAVM volume (r = 0.025, *p* = 0.78), mCCT (r = −0.014, *p* = 0.87), and the stasis index (r = −0.036, *p* = 0.69). The mCCT had very weak correlations with BAVM volume (r = −0.197, *p* = 0.02) and the stasis index (r = 0.041, *p* = 0.64). The median mCCT was significantly shorter in BAVMs with SSS drainage (2.09 s vs 2.75 s, *p* < 0.001) or parietal cortical drainage (1.91 s vs 2.59 s, *p* < 0.001) than in those without.

### Factors associated with symptomatic RICs

**Fig. 4:**
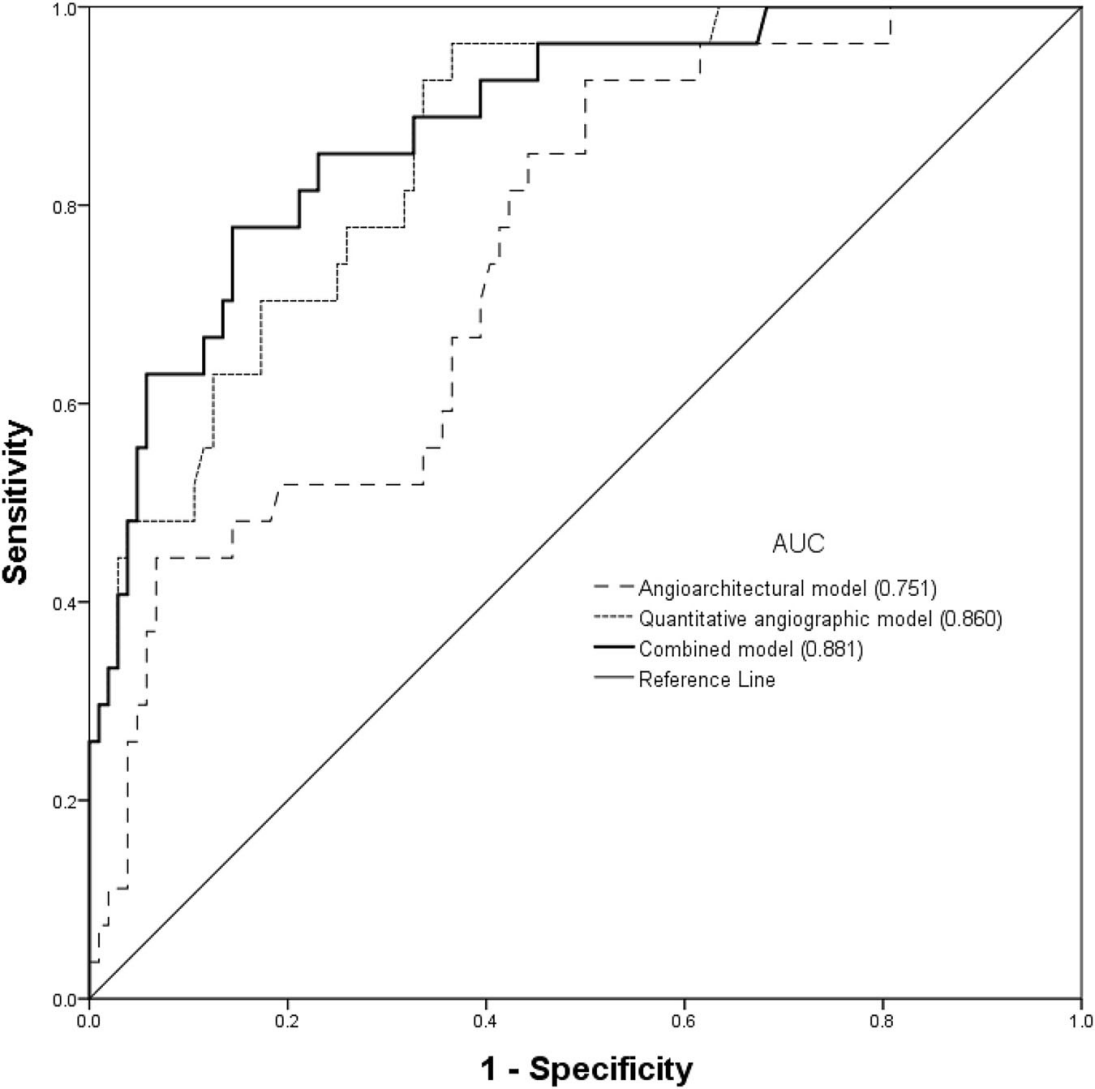
Receiver operating characteristic curves of angioarchitectural, quantitative angiographic, and combined models for symptomatic RICs. The areas under the curve (AUCs) were 0.751, 0.860, and 0.881 for the angioarchitectural, quantitative angiographic, and combined models, respectively

**Fig. 5:**
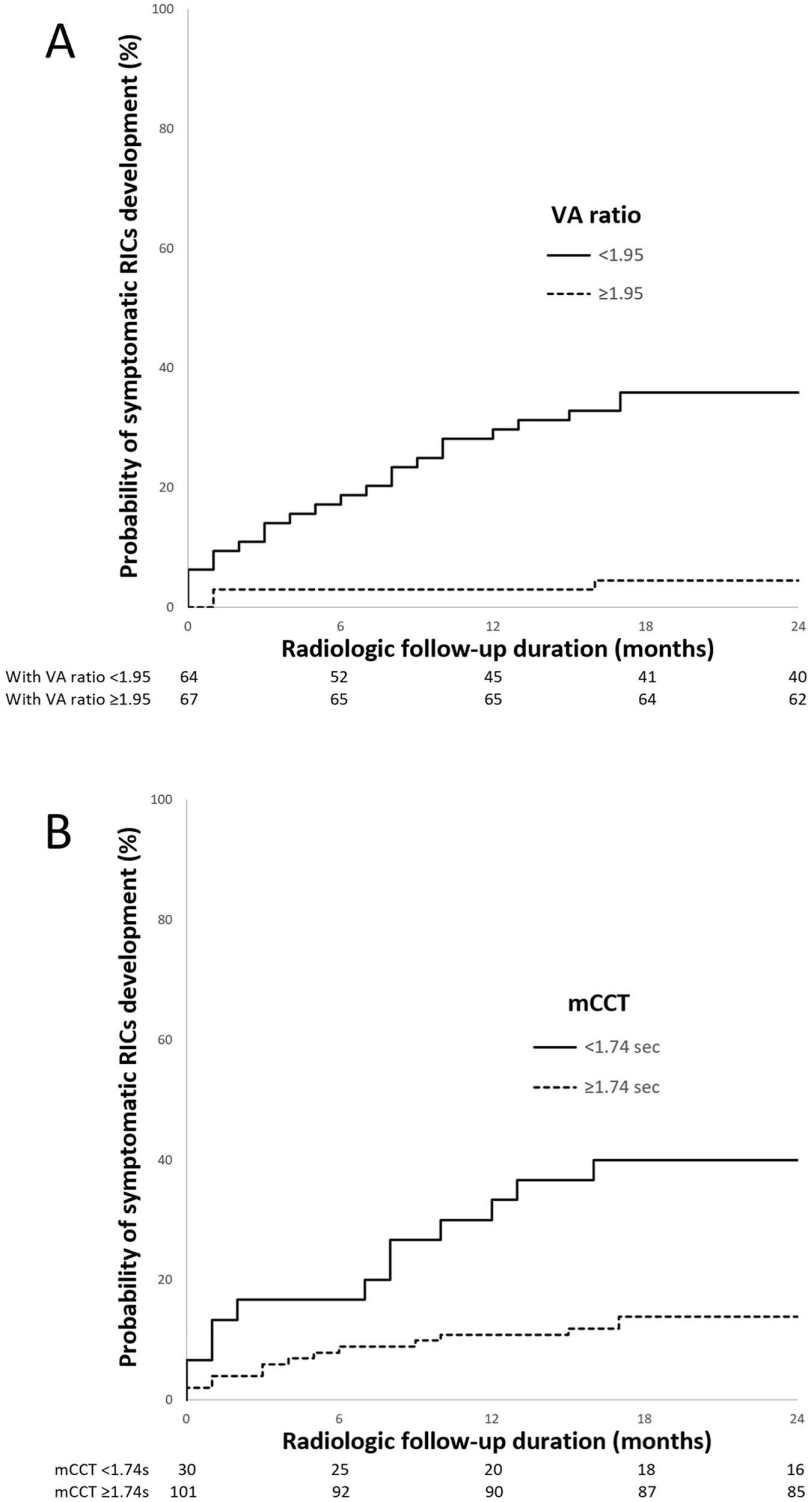
Actuarial probabilities of symptomatic RICs development within 24 months after GKRS, calculated on the basis of whether **A** the vein-artery (VA) ratio is ≥ 1.95 and **B** the mCCT is ≥ 1.74 s before GKRS. The estimated 24-month probability of symptomatic RICs development was 35.9 ± 6% and 6 ± 2.9% for VA ratio < 1.95 and ≥ 1.95, respectively. The estimated 24-month probability of symptomatic RICs development was 43.3 ± 9.0% and 13.9 ± 3.4% for mCCT < 1.74 s and ≥ 1.74 s, respectively. Values below the *x*-axis represent the number of patients remained in the analysis

Table 2 presents the results of univariable and multivariable Firth logistic regressions, which were performed to assess associations between BAVM characteristics and symptomatic RICs. Multivariable analysis indicated that a decreased VA ratio (odds ratio (OR): 2.377, *p* = 0.003) and decreased mCCT (OR: 1.944, *p* = 0.04) were independent factors. The cutoff values for differentiating patients with symptomatic RICs were a VA ratio of < 1.95 and mCCT of < 1.74 s. Table 3 presents the results of the angioarchitectural and quantitative angiographic models. In the quantitative angiographic model, a VA ratio of < 1.95 (OR: 14.69, *p* < 0.001) and mCCT of < 1.74 s (OR: 4.415, *p* = 0.02) were independent factors associated with symptomatic RICs. As displayed in Fig. 4, the quantitative angiographic model (area under curve (AUC) = 0.86; 95% confidence interval (CI): 0.789, 0.914) had a higher performance in association with symptomatic RICs than the angioarchitectural model did (AUC = 0.751; 95% CI: 0.668, 0.822), with an AUC difference of 0.109 (*p* = 0.02). Kaplan–Meier analysis revealed that patients with a VA ratio < 1.95 (χ^2^ = 18.276, *p* < 0.001) or mCCT < 1.74 s (χ^2^ = 13.064, *p* < 0.001) were more likely to develop symptomatic RICs (Fig. 5).

**Table 2:** Univariable and multivariable Firth logistic regression analysis of factors associated with the development of symptomatic RICs after GKRS

Variable	No. of patients	Univariable		Multivariable	
		OR (95% CI)	*p*-value	OR (95% CI)	*p*-value
Increased patient age, years	131	0.996 (0.97–1.024)	0.79		
Female	58	1.46 (0.633–3.37)	0.38		
Prior BAVM hemorrhage	59	0.453 (0.186–1.105)	0.08	0.457 (0.145–1.435)	0.18
Moderate RICs (RICs grade of ≥ II)	84	3.694 (1.254–10.884)	0.02	2.576 (0.724–9.172)	0.14
BAVM in thalamus or basal ganglia	14	2.457 (0.781–7.731)	0.12		
Deep location	32	1.141 (0.442–2.943)	0.79		
Eloquence	88	0.953 (0.395–2.302)	0.92		
BAVM volume > 5 cm^3^	82	3.009 (1.097–8.257)	0.03	1.37 (0.342–5.486)	0.66
Higher Spetzler–Martin grade	131	1.299 (0.818–2.061)	0.27		
Increased margin dose, Gy	131	0.725 (0.476–1.103)	0.13		
Angioarchitectural features					
Flow-related aneurysm	6	2.19 (0.439–10.91)	0.34		
Intranidal aneurysm	17	2.458 (0.843–7.166)	0.099	1.274 (0.351–4.621)	0.71
Neoangiogenesis	58	2.55 (1.08–6.022)	0.03	1.192 (0.369–3.845)	0.77
Deep venous drainage	50	0.641 (0.262–1.569)	0.33		
Venous stenosis	25	0.744 (0.244–2.266)	0.60		
Venous ectasia	27	1.508 (0.574–3.961)	0.40		
Pseudophlebitic pattern	22	3.48 (1.321–9.17)	0.01	2.639 (0.794–8.774)	0.11
Increased number of draining vein	131	1.31 (0.892–1.923)	0.17		
SSS drainage	83	2.894 (1.054–7.944)	0.04	1.095 (0.301–3.982)	0.89
Parietal cortical drainage	50	1.393 (0.599–3.24)	0.44		
Quantitative angiographic parameters					
Decreased VA ratio	131	2.218 (1.27–3.874)	0.005	2.377 (1.35–4.186)	0.003*
Increased transnidal time, s	131	1.22 (0.289–5.156)	0.79		
Increased transvenous time, s	131	1.115 (0.373–3.335)	0.85		
Decreased mCCT, s	131	2.069 (1.213–3.527)	0.008	1.944 (1.02–3.705)	0.04*
Increased average stasis index of distal draining veins	131	0.667 (0.285–1.559)	0.35		

*RICs* radiation-induced changes, *GKRS* Gamma Knife radiosurgery, *OR* odds ratio, *CI* confidence interval, *BAVM* brain arteriovenous malformation, *SSS* superior sagittal sinus, *VA ratio* vein-artery ratio, *mCCT* modified cerebral circulation time

* Statistically significant

**Table 3:** Angioarchitectural model and quantitative angiographic model of factors associated with the development of symptomatic RICs after GKRS

Variable	No. of patients	Angioarchitectural model		Quantitative angiographic model	
		OR (95% CI)	*p*-value	OR (95% CI)	*p*-value
Increased patient age, years	131	0.984 (0.951–1.019)	0.36	0.995 (0.959–1.033)	0.80
Female	58	1.904 (0.74–4.9)	0.18	1.838 (0.591–5.718)	0.29
Prior BAVM hemorrhage	59	0.519 (0.159–1.694)	0.28	0.299 (0.079–1.126)	0.07
BAVM in thalamus or basal ganglia	14	2.643 (0.554–12.606)	0.22	4.282 (0.841–21.813)	0.08
BAVM volume > 5 cm^3^	82	1.571 (0.387–6.382)	0.53	2.668 (0.602–11.829)	0.20
Increased margin dose, Gy	131	0.959 (0.545–1.688)	0.89	1.001 (0.518–1.932)	1.00
Intranidal aneurysm	17	1.626 (0.427–6.199)	0.48	…	…
Neoangiogenesis	58	1.272 (0.425–3.81)	0.67	…	…
Pseudophlebitic pattern	22	2.498 (0.795–7.843)	0.12	…	…
SSS drainage	83	2.545 (0.704–9.202)	0.15	…	…
VA ratio < 1.95	64	…	…	14.69 (3.978–54.255)	< 0.001*
mCCT < 1.74 s	30	…	…	4.415 (1.293–15.075)	0.02*

*RICs* radiation-induced changes, *GKRS* Gamma Knife radiosurgery, *OR* odds ratio, *CI* confidence interval, *BAVM* brain arteriovenous malformation, *SSS* superior sagittal sinus, *VA ratio* vein-artery ratio, *mCCT* modified cerebral circulation time

* Statistically significant

## Discussion

Several studies have investigated risk factors associated with symptomatic RICs (Table 4) [7–9, 11–14, 23, 24]. One of these studies reported a positive correlation between larger BAVM volume and symptomatic RICs development [11]. However, BAVMs > 5 cm^3^ were not independently associated with symptomatic RICs in our study. Yang et al indicated that volume coverage of brain parenchyma at a 12 Gy radiosurgical dose was an independent predictor of RICs in patients with unruptured BAVMs [25]. Furthermore, in the present study, the frequencies of grade II and III RICs were similar between patients with symptomatic and asymptomatic RICs. These findings suggest that factors other than perinidal edema may contribute to the development of symptomatic RICs. Three pathophysiological mechanisms of symptomatic RICs have been proposed: radiation injury, occlusive hyperemia, and upregulation of the vascular endothelial growth factor secondary to perinidal hypoxemia [8–10].

**Table 4:** Risk factors associated with the development of symptomatic RICs after GKRS in the literature

Authors [ref]	Symptomatic RICs rate (number of symptomatic RICs/all patients)	Risk factors associated with symptomatic RICs
Present study	20.6% (27/131)	Lower VA ratio and shorter mCCT
Grogan et al [12]	7.6% (15/197)	Deep pediatric BAVM location
Alzate et al [9]	16% (4/25)	Lower VA ratio and higher adverse radiation effect index
Hu et al [8]	17.9% (19/106)	BAVMs in brainstem or basal ganglia
Burke et al [23]	10.2% (55/539)	Pediatric BAVMs with prior embolization
Chen et al [14]	10.3% (55/536)	Unruptured pediatric BAVMs
Kano et al [11]	7.3% (55/755)	Larger BAVM volume, higher margin dose, a higher radiosurgery-based score, a higher Spetzler–Martin grade, and BAVMs in brainstem or thalamus
Ding et al [23]	9.6% (52/540)	Unruptured BAVMs
Oermann et al [24]	10.3% (50/484)	BAVMs without prior embolization
Yen et al [7]	8.6% (122/1426^a^)	Eloquent location and higher grade of RICs

*RICs* radiation-induced changes, *GKRS* Gamma Knife radiosurgery, *VA ratio* vein-artery ratio, *mCCT* modified cerebral circulation time, *BAVM* brain arteriovenous malformation

^a^ Counted in total procedures performed

Occlusive hyperemia involves a discrepancy between inflow and outflow that predisposes BAVMs to the development of venous congestion and regional parenchymal edema following GKRS [9, 10]. Alzate et al used the VA ratio to quantify the outflow drainage impairment of BAVMs and demonstrated an association between a lower VA ratio and RICs in 25 patients with BAVMs who underwent GKRS [9]. This association was also noted among the 131 patients included in the present study. Alzate et al also identified transit time between the nidus and draining veins as a predictor of RICs, although interobserver variability may have affected this finding [9, 26].

In our study, mCCT was used to quantify the hemodynamic effect of high-flow BAVM on cerebral circulation, and it achieved higher interobserver agreement than transnidal time did in a prior study [18]. Enhancing temporal resolution and incorporating quantitative flow measurements into DSA can improve hemodynamic studies of BAVMs [27]. An association has been reported between shortened cerebral circulation, as indicated by shorter mCCT in the present study, and seizure presentation and resistance to CO in BAVMs after GKRS [18, 20]. Increased nidus flow has commonly been observed in larger BAVMs and is capable of causing perfusion deprivation of the surrounding cerebral parenchyma due to arterial steal [28, 29]. Inadequate perfusion may cause chronic ischemic changes in the perinidal brain parenchyma, leading to neurological deficits [28]. In response to perinidal hypoxemia, vascular endothelial growth factor, hypoxia-inducible factor 1α, interleukin 6, metalloproteinase-9, and other cytokines may be upregulated and subsequently induce perivascular inflammation and neoangiogenesis [28, 30–32]. Subsequent neovascularization increases vessel wall permeability and radiation-induced inflammation in the adjacent brain tissue [8, 28]. Although BAVMs with SSS or parietal cortical drainage tended to have shorter mCCTs, decreased mCCT remained the independent factor associated with symptomatic RICs development after adjusting for SSS drainage. These findings suggest that mCCT shortening quantifies the impact of BAVM steal on the general cerebral circulation and may reveal the susceptibility of adjacent brain parenchyma to symptomatic RICs. Guo et al used perfusion MRI to show the elevated relative cerebral blood volume and relative cerebral blood flow ratios in the cerebral hemisphere ipsilateral to the BAVM [33], which may be quantitative measurements of perinidal perfusion disturbance potential for symptomatic RICs risk evaluation. Our study revealed that the general status of the hemisphere harboring BAVM, measured morphologically by the VA ratio and hemodynamically by the mCCT, could be more closely related to symptomatic RICs, rather than the specific hemodynamics for BAVM. By contrast, QDSA parameters focus on localized hemodynamics, namely the stasis index of the draining vein, which could be related to BAVM rupture [22]. A lower VA ratio and shorter mCCT may indicate the presence of occlusive hyperemia and neoangiogenesis, respectively, underlying symptomatic RICs. The present study noted that these two quantitative angiographic parameters may be objective imaging markers for risk estimation of symptomatic RICs rather than angioarchitectural analysis [8].

Loo et al found that BAVMs in patients with an mCCT of < 2.32 s exhibited resistance to CO after GKRS [18]. In our study, patients with an mCCT of < 1.74 s had a lower 60-month probability of BAVM CO than did those with an mCCT of ≥ 1.74 s (50 ± 9.9% vs. 71.3 ± 4.5%, *p* = 0.03). This finding suggests that the profound hemodynamic effects of high-flow BAVMs, indicated by an mCCT of < 1.74 s, may incur the risks of lower CO and higher symptomatic RIC rates after GKRS. The extent to which mCCT can guide therapeutic decision-making for patients with BAVMs requires further investigation.

Our study has several limitations. First, the single-center, retrospective study design entailed selection and information biases. Second, asymptomatic patients may have been lost to follow-up, causing overestimation of symptomatic RIC rates. In total, 44 (21.8%) of the 202 patients underwent < 2 years of MRI and clinical follow-up after GKRS and were excluded from analysis. Third, patients with infratentorial BAVMs or prior treatment history were also excluded because infratentorial BAVMs located in the brainstem are susceptible to symptomatic RICs development [8, 11]. Additionally, prior treatment may affect BAVM hemodynamics, compromising quantitative angiographic measurements. Therefore, our results should be applied with caution for different patient populations and treatment strategies.

## Conclusions

A lower VA ratio and shorter mCCT were independently associated with a higher risk of developing symptomatic RICs in patients with BAVMs who underwent GKRS. These two quantitative angiographic markers may indicate BAVMs with venous outflow impairment and high blood flow, respectively, facilitating the assessment of symptomatic RICs risk.

## Supplementary information

Electronic Supplementary Material

## Abbreviations

AUC: Area under curve
BAT: Bolus arrival time
BAVM: Brain arteriovenous malformation
CI: Confidence interval
CO: Complete obliteration
DSA: Digital subtraction angiography
GKRS: Gamma Knife radiosurgery
ICA: Internal carotid artery
mCCT: Modified cerebral circulation time
OR: Odds ratio
QDSA: Quantitative DSA
RICs: Radiation-induced changes
ROC: Receiver operating characteristic
ROI: Region of interest
SSS: Superior sagittal sinus
VA: Vein-artery
