# Supplementary material for: Quantitative angiographic markers associated with symptomatic radiation-induced changes in supratentorial brain arteriovenous malformations after radiosurgery
Source: Eur Radiol. 2025 Jul 31;36(2):901–15. doi: 10.1007/s00330-025-11884-8 (PMC12953479; doi:10.1007/s00330-025-11884-8)
Supplement: Supplementary file 1 — Electronic Supplementary Material [file 330_2025_11884_MOESM1_ESM.pdf]

Quantitative angiographic markers associated with symptomatic radiation-induced changes in supratentorial brain arteriovenous malformations after radiosurgery

Electronic Supplementary Material

Measurement of the supplying artery diameters in a brain arteriovenous malformation (BAVM) with multiple arterial pedicles

The supplying arteries were defined as the trunk arteries giving rise to the arterial pedicles feeding the BAVM nidus, which theoretically represent the cumulative hemodynamic effect of the branching vessels. All individual supplying arteries were measured, when possible, on both anteroposterior (**Supplementary Figure 1a**) and lateral (**Supplementary Figure 1b**) views of digital subtraction angiography (DSA) images, avoiding overlapping segments. To ensure precision and minimize errors, images were magnified until the arterial edges were clearly visualized (**Supplementary Figure 1c**). If discrepancies arise between measurements of the same artery on anteroposterior and lateral views, the larger value would be recorded. Under our standardized protocol, the interobserver agreement between the two neuroradiologists for vein-artery ratio was moderate (intraclass correlation coefficient: 0.56).[1]

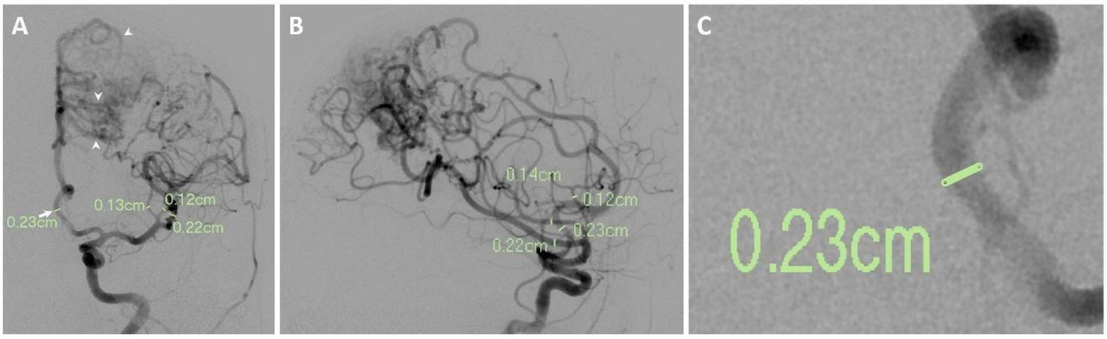

Supplementary Figure 1. Illustration of measurements of supplying artery diameter in a BAVM with multiple arterial pedicles on anteroposterior **(a)**, lateral **(b)**, and magnified **(c)** views of DSA images. **(a)** If distinguishing and

measuring each artery individually is impractical due to numerous overlapping arterial pedicles (arrowheads), we would measure their common trunk (arrow).

Reference:

1. Loo JK, Hu YS, Kao WL et al (2024) Shortened Cerebral Circulation Time Predicts Resistance to Obliteration in High-Flow Brain Arteriovenous Malformations After Stereotactic Radiosurgery. Neurosurgery 95:1429-1440
